# Joint deep learning for batch effect removal and classification toward MALDI MS based metabolomics

**DOI:** 10.1186/s12859-022-04758-z

**Published:** 2022-07-10

**Authors:** Jingyang Niu, Jing Yang, Yuyu Guo, Kun Qian, Qian Wang

**Affiliations:** 1 School of Biomedical Engineering, Shanghai Jiao Tong University, Shanghai, 200030 China; 2 School of Biomedical Engineering, ShanghaiTech University, Shanghai, 201210 China

**Keywords:** Metabolomics, Deep learning, Batch effect, Diagnostic accuracy

## Abstract

**Background:**

Metabolomics is a primary omics topic, which occupies an important position in both clinical applications and basic researches for metabolic signatures and biomarkers. Unfortunately, the relevant studies are challenged by the batch effect caused by many external factors. In last decade, the technique of deep learning has become a dominant tool in data science, such that one may train a diagnosis network from a known batch and then generalize it to a new batch. However, the batch effect inevitably hinders such efforts, as the two batches under consideration can be highly mismatched.

**Results:**

We propose an end-to-end deep learning framework, for joint batch effect removal and then classification upon metabolomics data. We firstly validate the proposed deep learning framework on a public CyTOF dataset as a simulated experiment. We also visually compare the t-SNE distribution and demonstrate that our method effectively removes the batch effects in latent space. Then, for a private MALDI MS dataset, we have achieved the highest diagnostic accuracy, with about 5.1 ~ 7.9% increase on average over state-of-the-art methods.

**Conclusions:**

Both experiments conclude that our method performs significantly better in classification than conventional methods benefitting from the effective removal of batch effect.

**Supplementary Information:**

The online version contains supplementary material available at 10.1186/s12859-022-04758-z.

## Background

Metabolomics hunts for quantitative descriptions of complex biological samples, and associates clinical observations of diseases with temporal fluctuations of metabolites. By measuring and modelling metabolism alternations in biological samples, metabolomics offers much insight into the effects of diet, diseases, and therapies [1, 2]. Many novel metabolomics technologies emerge and can now profile big quantity of data effectively. For example, matrix assisted laser desorption/ionization mass spectrometry (MALDI MS) [3] offers fast processing speed (~ seconds) and low sample consumption (~ μL). Meanwhile, cytometry by time-of-flight (CyTOF), a novel single-cell analysis technology, extends the data dimensionality to simultaneously measuring 40 + cellular parameters [4]. It has thus been applied to track the expression levels of biomarkers that reflect various cellular attributes [5].

While non-targeted metabolomics has the capability of encoding complex biological samples, it is often a must to use sophisticated data interpretation techniques to facilitate clinical applications. Many conventional studies establish statistical differences at the population level. However, it is not easy to generalize their findings to the sample or subject level, for the sake of individualized diagnosis and treatment [6]. Concerning the rapid progress of machine learning and especially deep learning in past years, there is a trend to adopt these advanced tools to search for biomarkers from non-targeted metabolomics data and then establish data-driven disease models that are applicable to individual patients [7–9].

A major challenge in generalizing a machine learning model to real-case metabolomics data points to batch effect, which is almost inevitable to occur. Exactly, batch effect accounts for the measurements that behave differently across experimental conditions yet are unrelated to the biological variables of interest under consideration [10]. The origin of batch effect is far-ranging, including different platforms, different reagents of the same sample, and different time points to acquire data, etc. In MALDI MS, for example, batch effect (if not calibrated) might lead to inconsistent diagnosis, if the serum sample for a patient was repeatedly processed in different MS target plates. Therefore, it is necessary to suppress the batch effect in these metabolomics scenarios.

The rattling bottleneck of batch effect has drawn intense researches in the past. There are two conventional ways to suppress batch effect, i.e., location-scale (LS), and matrix-factorization (MF) [11]. For example, ComBat [12] is a popular LS method. It employs a Bayesian framework to model the data, by parameterizing location and scale for each batch and each feature independently [13]. Other LS approaches, including distance-weighted discrimination (DWD) [14], one-way analysis of variance (ANOVA) approach [15] and Ratio_G [16], assume normal data distribution for each batch and align the distributions of different batches accordingly. However, the assumption in the LS methods may be over-simplified to treat complex batch effect as additive and multiplicative components.

As an alternative to the LS methods, surrogate variable analysis (SVA) provides an MF way to remove batch effect [17]. The MF approaches assume that the data variation induced by batch effect is independent with the target labels. In this way, the data can be factorized into two parts, corresponding to the distortion of batch effect and the left-out part [11, 17]. However, the modeling in the MF approaches relies on the assumption on the independence of batch effect and data labels, which may not always be valid in practice.

Frequently, in the field of non-targeted metabolomics diagnosis, there is a need to construct a discriminative model that can train a batch of source data and apply it to predict the labels for target batch data. The Ratio_G method [16] adjusts data for enhancement of label prediction. The fSVA method [13] is also capable of predicting the labels for unseen samples. In fSVA, SVA is first used to calibrate batch effect in the training dataset. Then, the probability weights and coefficients estimated on the training dataset are utilized to remove batch effect in the new test samples. The classifier trained on the calibrated dataset can finally be applied for prediction. However, the matrix factorization may sometimes reduce the data variation attributed to batch effect at the cost of decreasing the discrepancy between the disease group and the controls, which in turn lowers the classification accuracy in subsequent analysis [13].

Computational analysis of high-throughput omics data that refers to genomics, transcriptomics, proteomics, metabolomics and radiomics has become popular in recent decades [7]. Considering many measurements (corresponding to feature dimensionality) and usually small numbers of samples (or sample size), it is obviously a challenge to machine learning [8]. The recent leap of deep learning, on the other hand, provides an unprecedented tool to conquer those obstacles. Different kinds of deep learning architectures, such as convolutional neural network (CNN) [18], recurrent neural network (RNN) [19], long-/short-term memory (LSTM) [20], autoencoder (AE) and generative adversarial network (GAN) [21], have been applied in various omics studies. It has outperformed many conventional machine learning techniques, e.g., at breast cancer classification [22], automatic glaucoma detection [23], human gait recognition [24] and intelligent fusion-assisted skin lesion localization and classification [25]. All of these analyses can potentially help physicians to provide precise diagnosis and individualized treatment.

With the popularity of deep learning, Shaham et al. [26] used ResNet to remove batch effect. While deep learning has powerful capability of approximating highly nonlinear mapping, the solution in Shaham et al. is unsupervised in nature (i.e., without knowing the disease labels the of samples). On the other hand, the GAN based NormAE [27] constructs an adversarial training procedure between a nonlinear AE to remove batch effect and a discriminator to classify the batch labels based. The discriminative power of the learned networks is critical to diagnostics and identification of metabolic biomarkers. Unfortunately, by only reducing the mismatching across different batches, the diagnosis efficacy would not necessarily improve.

The subsequent learning-based classification and diagnosis can benefit only if the batch effect among sample data is properly handled. To address the above issues, we propose a joint deep learning framework to calibrate batch effect first and then conduct sample classification (e.g., to derive disease diagnosis). Our framework consists of three major networks that interact with each other closely: (1) Given individual input batches of metabolomics data, we pass them through the calibrator such that they are aligned in the latent feature space; (2) A subsequent discriminator derives from the latent space, supervised by the known labels of certain batch in training, and completes classification for the other test batch; (3) The reconstructor(s) also derives from the latent feature space and restores all input batches, to ensure that the input batches are well learned throughout the networks. We first conduct a set of simulation experiments on the public CyTOF data to verify the effectiveness of our method. Next, we apply the proposed method to our private MALDI MS data, and demonstrate superior performance in achieving not only good batch effect removal but also satisfactory classification capability.

## Results

In this study, we propose a deep learning framework to remove batch effect from MALDI MS based metabolomics data. To verify our framework, we first conduct a set of simulation experiments on a public dataset using high-throughput technology of CyTOF. Next, we report experimental results aiming at our private MALDI MS data and compare it to several representative methods in the literature. Detailed evaluations are reported in the next.

### Evaluation metrics

Our evaluation focuses on batch effect removal and classification performance, respectively. Particularly, for batch effect removal, we adopt MMD as a quantitative metric. We also turn to t-SNE, which is a popular dimension reduction tool, to visualize the distribution of the high-dimensional data. For classification performance, we adopt four metrics on the test set, including Accuracy (ACC), F_score, Area Under Curve (AUC), and Matthews correlation coefficient (MCC) [16, 28].

### Simulation study on public CyTOF data

#### Dataset

CyTOF is a mass cytometry technology that allows simultaneous measurements of multiple biomarkers in each cell of a specimen [29]. We aim to validate the capability of our method with a subset of the publicly available data used in [26], which originally derived from Finck et al. [30]. In particular, Peripheral Blood Mononuclear Cells (PBMCs) were collected from two sclerosis patients, and thawed on two different days that corresponded to two batches naturally. The classification labels are specified as being incubated with (positive) or without (negative) ionomycin marks that represent different mass cytometry antibodies. Thus, we can specify Day 1 (Batch 1) of Patient 1 for training and Day 2 (Batch 2) for testing, and so on for Patient 2. There are 1858 (Day 1 of Patient 1), 1460 (Day 2 of Patient 1), 4308 (Day 1 of Patient 2), 3530 (Day 2 of Patient 2) samples per batch, while each sample has 25 measurements or features. In this data, we focus on the batch effect (Days) while the two patients are independently considered.

#### Validation of batch effect removal

We first perform quantitative evaluation by computing the MMD between the source and target batches, before and after calibration. Note that to derive each MMD value in Table 1, we compute from a subset of 500 samples randomly drawn from all samples available and then take the average over 10 permutation runs. Particularly, we also compute the in-batch MMD in the same way, which represents the lower-bound of the MMD measure as batch effect doesn’t exist inside a batch presumably. As shown in Table 1, our calibration decreases the MMD value between the two batches (i.e., 0.067 ± 0.005 for Patient 1 after being processed by our method, 0.092 ± 0.005 for Patient 2, both of which are lower than the raw data and the results calibrated by other methods). Meanwhile, the MMDs produced by our approach become closer to the in-batch limits, which are listed as the last two columns of the table. This demonstrates our framework can suppress batch effect effectively.

**Table 1:** MMDs of the CyTOF data before and after being calibrated by individual methods

	Raw	ComBat	Ratio_G	fSVA	ResNet	NormAE	Ours	In Batch 1	In Batch 2
Patient 1	0.243 ± 0.010	0.167 ± 0.006	0.243 ± 0.010	–	0.116 ± 0.008	0.113 ± 0.005	0.067 ± 0.005	0.053 ± 0.005	0.057 ± 0.005
Patient 2	0.230 ± 0.010	0.139 ± 0.005	0.230 ± 0.010	0.158 ± 0.007	0.120 ± 0.009	0.131 ± 0.007	0.092 ± 0.005	0.065 ± 0.006	0.064 ± 0.005

“Raw” represents the MMD value of the source and target batches before calibration. “—” means that the fSVA method has collapsed due to numerical singularity in our computation. “In Batch 1 (or 2)” represents the intrinsic MMD without being corrupted by batch effect inside each batch

**Fig. 1:**
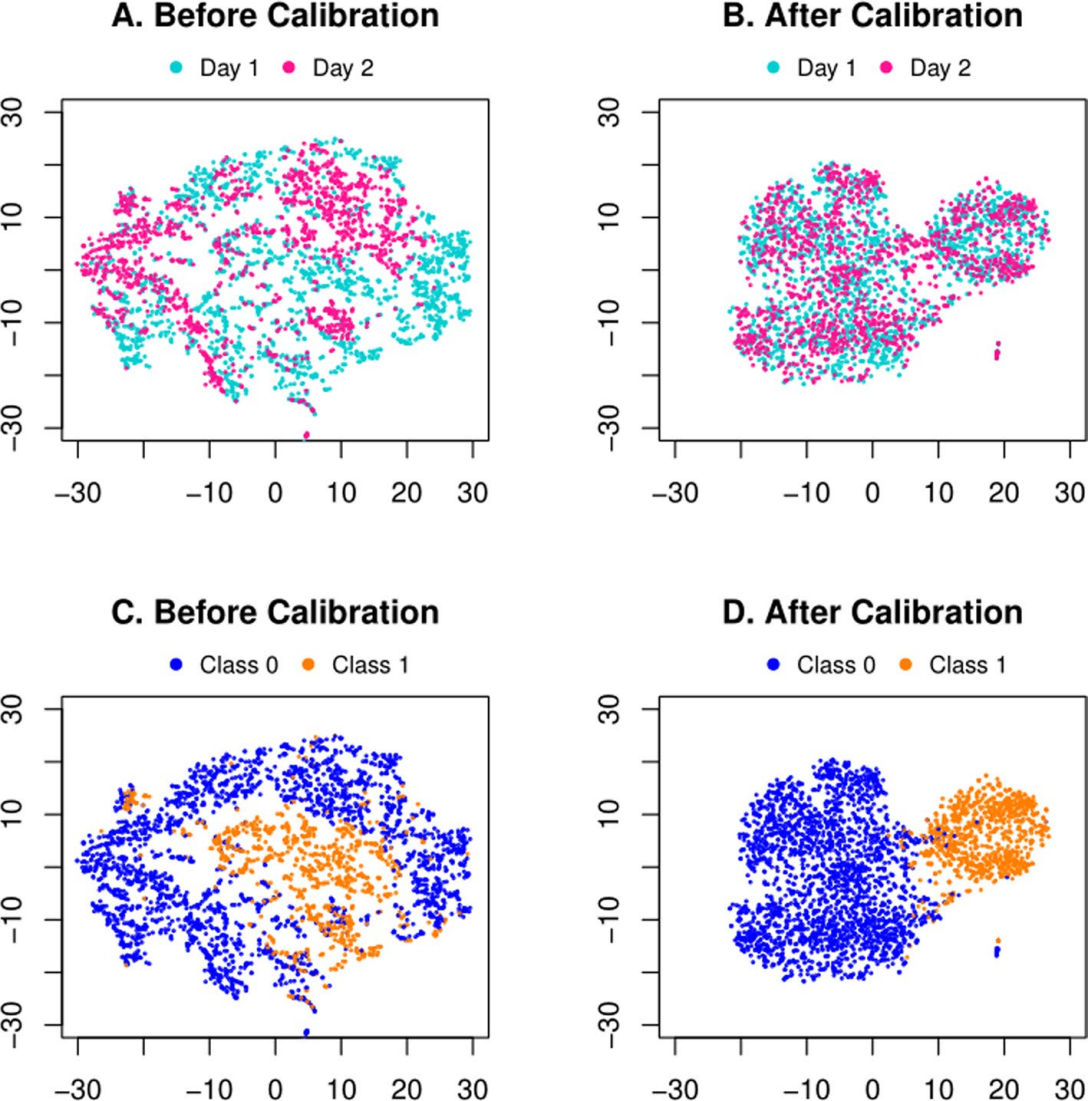
Visualization of the public CyTOF data of Patient 1. In (**A**) and (**B**), different colors highlight the two batches (Days). In (**C**) and (**D**), different colors identify true labels of the samples

We further list a common and straightforward visualizing comparison before and after batch effect removal. Figure 1 shows t-SNE plot of the raw inputs ${\mathbf{X}}_{1}$, ${\mathbf{X}}_{2}$ and the calibrated data ${\mathbf{Z}}_{1}$, ${\mathbf{Z}}_{2}$ for Patient 1. Notice that, we have the same t-SNE axes to span the latent space for all four figures. Ideally, the samples of the same classification label should be close to each other in the feature space, instead of distributing with respect to the batch factor. However, as in Fig. 1A, one may observe that the two batches (Days, in different colors) are distributed in different patterns, suggesting a clear batch effect that separates them and may hinder subsequent classification (c.f. Figure 1C, colored in accordance to sample labels before calibrating batch effect). On the contrary, after being calibrated as in Fig. 1B, the two batches share the distributions that are fully entangled, implying their in-between mismatch due to batch effect is removed. Meanwhile, as in Fig. 1D, the samples are naturally grouped into two halves in accordance to their true labels other than batches. This is partially due to our discriminator in the network, which helps shape the feature space to not only remove batch effect but also facilitate the classification of the labels.

#### Classification performance

The contribution of our framework can further be validated by the classification performance quantitatively. There are two possible classification tasks, i.e., training with Batch 1 (Day 1) and testing on Batch 2 (Day 2), or training with Batch 2 (Day 2) and testing on Batch 1 (Day 1). As shown in Table 2, the quantitative results indicate that our method can improve in both tasks, after the two batches are processed through batch effect calibration and then classification.

**Table 2:** Classification results on the public CyTOF data

	Source	Target			
		Before calibration		After calibration	
		Day 1	Day 2	Day 1	Day 2
*Patient 1*					
ACC	Day 1	0.962	0.939	0.962	0.951
	Day 2	0.947	0.961	0.964	0.961
F-score	Day 1	0.931	0.885	0.931	0.909
	Day 2	0.907	0.931	0.935	0.931
AUC	Day 1	0.962	0.911	0.962	0.928
	Day 2	0.958	0.961	0.968	0.961
MCC	Day 1	0.906	0.845	0.906	0.877
	Day 2	0.875	0.904	0.911	0.904
*Patient 2*					
ACC	Day 1	0.985	0.973	0.985	0.975
	Day 2	0.973	0.982	0.978	0.982
F-score	Day 1	0.939	0.901	0.939	0.905
	Day 2	0.895	0.934	0.908	0.934
AUC	Day 1	0.976	0.951	0.976	0.937
	Day 2	0.963	0.966	0.947	0.966
MCC	Day 1	0.931	0.885	0.931	0.891
	Day 2	0.881	0.924	0.895	0.924

When the source and target indices are the same, the reported metrics are for the in-batch classification by tenfold cross-validation

The classification performance can even be comparable to the case when batch effect is theoretically ruled out. Particularly, we examine the in-batch classification performance by conducting tenfold cross-validation within a certain batch. These results are perceived as a reference of the classification performance without being corrupted by batch effect, which are listed in the diagonals of Table 2 when the source and target indices are the same. Our proposed method produces the metrics that are not only close to the in-batch classification performance, but also can sometimes exceed them, which will be discussed later.

### Real-case study on private MALDI MS data

#### Dataset

The method we have developed is mainly aimed at serum MALDI MS dataset. All healthy controls (HCs) and systemic lupus erythematosus (SLE) patients were recruited from Renji Hospital, Shanghai Jiao Tong University School of Medicine. The SLE patients were diagnosed according to the criteria of 2012 Systemic Lupus International Collaborating Clinics (SLICC) [31], and the healthy controls showed no symptoms of rheumatic disease or other disease. All the participants have provided the informed consents for this study. In summary, we have a dataset of 598 subjects (306 SLE patients, and 292 HCs). Based on the limitation of sample volume that the MS target plates could hold, all subjects are divided into three plates (and thus batches), each of which has 201 (94 SLEs, 107 HCs), 212 (120 SLEs, 92 HCs), and 185 (92 SLEs, 93 HCs) subjects, respectively. The task is to diagnose SLE patients from HCs based on the MALDI MS data.

The collection process for the MALDI MS data was early introduced in [32]. Quality control (QC) was enforced to ensure that the data acquired are of high quality and reproducible. In particular, the mass calibration was conducted using standard molecules to ensure the precise mass measurement and avoid intra-plate deviation. For each subject, we repeated LDI MS detection for five times to enhance reproducibility and stability. Only MS signals with a signal-to-noise ratio over 3 and the relative standard deviation (RSD) more than 5% were used for the identification of molecules. Therefore, each subject would have 1–5 (mostly 5) samples in the final. In total, for the three batches, we collected 1005 samples for 201 subjects, 1053 samples for 212 subjects, and 925 samples for 185 subjects, respectively. All data was preprocessed through smoothing, baseline correction, peak extraction, alignment and normalization, following the protocol in [32]. For each sample, the m/z range was set from 100 to 1000 and 814 features were finally obtained after data preprocessing.

#### Validation of batch effect removal

Similar to the experiment on the public CyTOF data, we also compute the MMDs between every pair of source and target batches on the MALDI MS dataset. As shown in Table 3, our calibration decreases the MMDs to large extent (i.e., after being processed by our approach, 0.071 ± 0.008, 0.072 ± 0.007, 0.073 ± 0.008, 0.069 ± 0.006, 0.074 ± 0.008, 0.070 ± 0.007 for six source/target combinations of three batches, respectively, all of which are lower than the raw data and the calibrated results by other algorithms). Meanwhile, the MMDs produced by our framework are closest to the in-batch measures, which are listed in the last column of the table and can be perceived as MMD bounds of batch effect removal.

**Table 3:** MMDs of the SLE data before and after being calibrated by individual methods

Source	Target	Raw	ComBat	Ratio_G	fSVA	ResNet	NormAE	Ours	In Source
1	2	0.217 ± 0.010	0.125 ± 0.008	0.217 ± 0.009	0.285 ± 0.013	0.153 ± 0.010	0.202 ± 0.012	0.071 ± 0.008	0.053 ± 0.005
	3	0.696 ± 0.016	0.134 ± 0.009	0.292 ± 0.008	0.351 ± 0.014	0.144 ± 0.008	0.166 ± 0.008	0.072 ± 0.007	
2	1	0.217 ± 0.010	0.125 ± 0.008	0.221 ± 0.010	0.214 ± 0.011	0.153 ± 0.010	0.202 ± 0.012	0.073 ± 0.008	0.062 ± 0.005
	3	0.623 ± 0.016	0.147 ± 0.009	0.245 ± 0.010	0.242 ± 0.010	0.176 ± 0.010	0.145 ± 0.008	0.069 ± 0.006	
3	1	0.696 ± 0.016	0.134 ± 0.009	0.291 ± 0.008	0.289 ± 0.012	0.144 ± 0.008	0.166 ± 0.008	0.074 ± 0.008	0.064 ± 0.004
	2	0.623 ± 0.016	0.147 ± 0.009	0.240 ± 0.010	0.223 ± 0.008	0.176 ± 0.010	0.145 ± 0.008	0.070 ± 0.007	

The first two columns present the data combinations of different batches participating in the comparison. The other columns are similar to the comparisons in Table 1

**Fig. 2:**
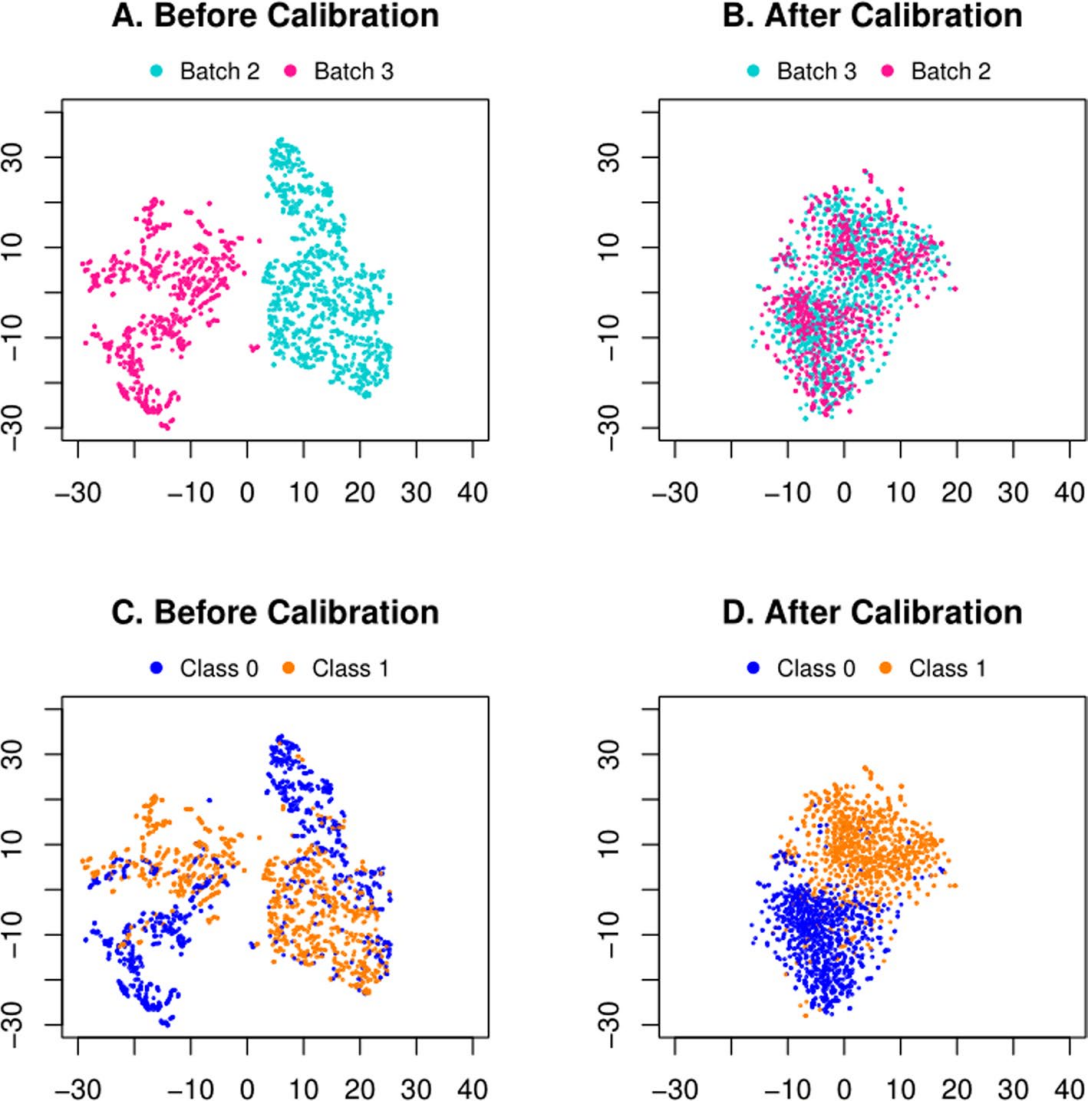
Visualizations of Batch 3 as the source and Batch 2 as the target of the private MALDI MS data. **A** and **B** are colored by batch indices. In (**C**) and (**D**), the samples are colored by disease labels (Class 1: SLE; Class 0: control)

We also visualize the data distribution, by projecting the raw data and the calibrated data to the same feature space by t-SNE. We have particularly chosen Batch 3 as the source and Batch 2 as the target in Fig. 2 for demonstration. Notice that we have the same t-SNE feature space across all plots. The two selected batches (ID = 3 and 2) are completely mixed together (Fig. 2B) compared with the case before calibration (Fig. 2A), which confirms that the calibration suppresses the batch effect. Meanwhile, if colored by the disease labels (SLE vs. control), one may notice that the samples are barely separable before calibration (Fig. 2C), yet much clearly separable after calibration (Fig. 2D). The remaining five combinations of source/target batches are listed in Figure S2 of Additional file 1, where all distributions are rendered in the same t-SNE feature space.

**Fig. 3:**
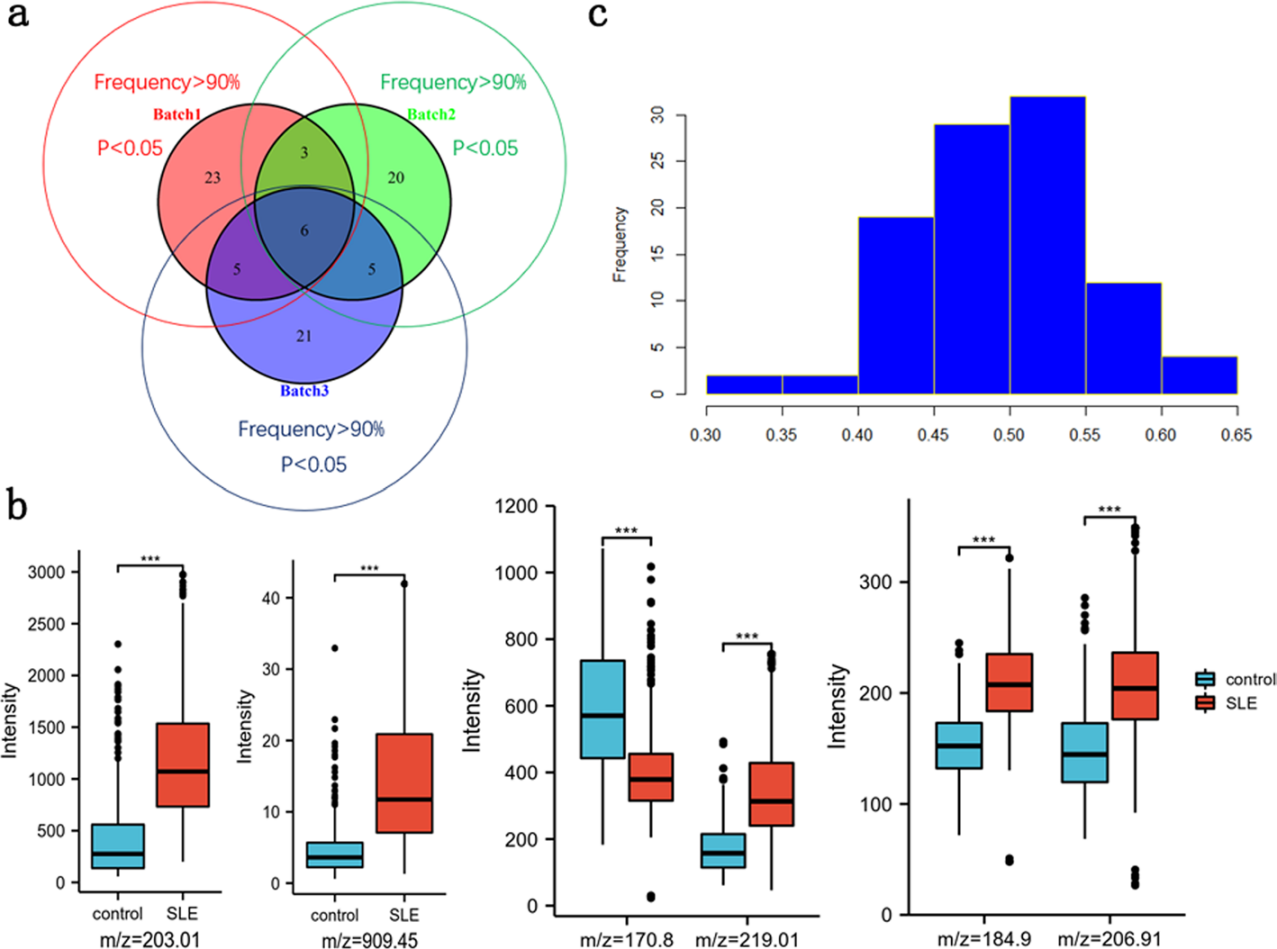
**a** Venn diagram about the number of metabolite peak intersections within three batches as potential biomarkers with model selection frequency > 90% and p < 0.05. **b** Boxplots of six common m/z features that reflect significant differences for the case and control groups. **c** Permutation test of random labels for batch 1 as source and batch 2 as target

An additional objective of non-targeted metabolomics is to find putative biomarkers to unravel the differences in the molecular underpinnings between two metabolic states, i.e., phenotypes. To this end, we have found 37, 34, and 37 potential metabolic biomarkers with model selection frequency > 90%, *p* < 0.05 in each batch, respectively (Fig. 3a). Then we select six common features to display the significant differences in the expression levels of the SLE and healthy groups, as shown in Fig. 3b. Taking batch 2 as an example, since the expression abundance of these m/z features is quite different from others, we divide them into several groups to better present the differences of case and control.

#### Diagnosis performance

We then evaluate the classification performance in a quantitative way. As shown in Table 4, given a single source batch for training and another single target batch for test, one can notice that the classification performance has substantially increased after batch effect removal by our framework. For example, when Batch 1 is used as the training set and Batch 2 for test, the metrics of ACC, F_score, AUC, and MCC have increased by 13.6%, 9.7%, 15.3% and 26.9%, respectively. Meanwhile, other source/target combinations of batches have also achieved improvement after the batch effect in raw data is calibrated. The above results prove that our framework can remove batch effect effectively, leading to superior diagnostic performance.

**Table 4:** Classification results on the MALDI MS data

	Source	Target					
		Before calibration			After calibration		
		1	2	3	1	2	3
*Sample*							
ACC	1	0.926	0.753	0.813	0.926	0.889	0.879
	2	0.799	0.911	0.828	0.875	0.911	0.870
	3	0.876	0.763	0.927	0.907	0.884	0.927
F-score	1	0.915	0.807	0.828	0.915	0.904	0.875
	2	0.814	0.919	0.823	0.867	0.919	0.863
	3	0.865	0.741	0.929	0.904	0.899	0.929
AUC	1	0.923	0.729	0.813	0.923	0.882	0.879
	2	0.809	0.912	0.828	0.875	0.912	0.870
	3	0.874	0.786	0.927	0.909	0.879	0.927
MCC	1	0.857	0.505	0.637	0.857	0.774	0.758
	2	0.648	0.831	0.656	0.749	0.831	0.743
	3	0.750	0.593	0.866	0.816	0.764	0.866
*Subject*							
ACC	1	0.922	0.769	0.822	0.922	0.896	0.892
	2	0.791	0.909	0.832	0 .891	0.909	0.876
	3	0.871	0.769	0.925	0.921	0.892	0.925
F-score	1	0.911	0.821	0.837	0.911	0.910	0.890
	2	0.814	0.918	0.827	0.883	0.918	0.870
	3	0.862	0.749	0.926	0.916	0.905	0.926
AUC	1	0.919	0.744	0.822	0.919	0.889	0.892
	2	0.802	0.909	0.832	0.890	0.909	0.875
	3	0.870	0.793	0.925	0.921	0.888	0.925
MCC	1	0.848	0.539	0.658	0.848	0.789	0.784
	2	0.636	0.827	0.666	0.780	0.827	0.753
	3	0.740	0.608	0.858	0.841	0.779	0.858

The top half is conducted in the sample level, and the bottom half in the subject level. When the source and target IDs are the same, we perform in-batch cross-validation, whose results are free of batch effect

While the in-batch diagnostic prediction is performed by tenfold cross-validation, we observe that our method produces the results that get much closer to the ceilings where batch effect is completely ruled out. Furthermore, we also conduct random permutation test to see weather random or “fake” labels could achieve good results or not. Taking batch 1 for training and batch 2 for test as an example, our implementation is to randomly shuffle the labels of training set 100 times and count the frequency of accuracy (ACC) value in each interval. As shown in Fig. 3c, the ACC values are distributed in a bell shape and the overall result of random labels is really poor. It turns out that the random label generation is reasonable and our model is practical.

It is worth noting that, in our MALDI MS data, each subject comes with multiple samples during data acquisition. While each sample can have a predicted label in test, the final diagnosis should be ensembled to the subject level. Specifically, we can infer the classification label per sample, and then derive the classification result for the subject as the median of all samples in one subject [32]. The subject-level diagnosis results are reported in Table 4. Similar to the sample-level evaluation, we find that the sample-level prediction can reflect the ground-truth diagnoses of the subjects effectively. Note that the following comparisons with other methods are all reported in the sample level.

#### Comparison with state-of-the-art methods

We select several popular tools including ComBat [12], Ratio_G [16], fSVA [13], ResNet [26] and NormAE [27] for further comparison. Accuracy of cross-batch prediction in the sample level is used to evaluate effectiveness of the methods, as it reflects a common circumstance for the classification purpose. To achieve fair comparison, we evaluate their performance based on the same processing pipeline and by following recommended protocols in their reports.

The comparing results are reported in Table 5. The performance of ours is superior over all other methods. On average, the accuracy of our framework is 5.1 ~ 7.9% higher than other methods.

- Compared with ComBat, the popular LS method for population-level correction, our method has improved by 7.3% (from 81.1% to 88.4%).
- Ratio_G is a typical LS method for batch effect removal. However, the method in overall (81.9%) is inferior to ours (88.4%) by a margin 6.5%. Their relatively poor performance is attributed to the linear nature of simple addition and multiplication being superimposed.
- The representative MF method of fSVA performs slightly better than other algorithms, but it is still below ours, e.g., especially 77.3% vs. 88.9% in Table 5 for the first source/target combination.
- Compared with ResNet, the first deep learning algorithm used for batch effect removal, our method has also achieved 6.0% improvement (82.4% vs. 88.4% in overall) taking advantage of label supervision from the source data.
- NormAE is the latest strategy that adopted the popular adversarial network in recent years. Even though, it still lags behind ours by 5.1%, which is trapped in the idea of traditional GAN that distinguishes domain labels rather than true biological labels directly during the adversarial training.

**Table 5:** Comparison of diagnosis accuracy with one source batch for training and another target batch for testing

Source	Target	Baseline	ComBat	Ratio_G	fSVA	ResNet	NormAE	Remove_R	Ours
1	2	0.753	0.778	0.798	0.773	0.791	0.805	0.852	**0.889**
1	3	0.813	0.797	0.858	0.836	0.803	0.812	0.856	**0.879**
2	1	0.799	0.817	0.821	0.857	0.824	0.827	0.839	**0.875**
2	3	0.828	0.851	0.818	0.829	0.852	0.866	0.833	**0.870**
3	1	0.876	0.861	0.864	0.854	0.868	0.889	0.863	**0.907**
3	2	0.763	0.759	0.754	0.824	0.805	0.799	0.821	**0.884**
Overall		0.805	0.811	0.819	0.829	0.824	0.833	0.844	**0.884**

“Baseline” denotes classification based on raw input data without any calibration for batch effect removal

Particularly, the first combination (Batch 1 as source, Batch 2 as target) yields the most improvement for our method (9.1%) compared to the second-ranking approach (Ratio_G). All methods perform well in the fifth combination (Batch 3 as source, Batch 1 as target) partially due to their intrinsic data distribution. These results in overall indicate that our approach not only removes batch effect more effectively than other methods, but also achieves classification and diagnosis more accurately.

## Discussion

Non-targeted metabolomics is considered as a rapid, accurate and noninvasive technique, especially MALDI MS, and it is becoming an increasingly popular tool in discovering diagnostic biomarkers of diseases. However, batch effect is ubiquitous in these types of high-throughput metabolomics informatics. For instance, to facilitate large-scale MS experiments and allow for data analysis, a batch adjustment process is required to reduce variability among these data. If one was not aware of it, subsequent experiments would lead to incorrect conclusions.

In this study, our primary goal is to use existing (source) data to create a model to predict the class labels for future (target) data. Thus, we formulate our task as an integration of both batch effect removal and classification, which makes our task significantly different with the existing works. To this end, we introduce a joint deep learning framework for batch effect removal and classification toward MALDI MS based metabolomics. Our main contributions include: (1) the calibrator-reconstructor design instantiating the encoder-decoder pathway, such that all batches well preserve their intrinsic data patterns throughout self-learning; (2) the discriminator, which also follows the calibrator to remove batch effect, can classify input samples accordingly.

We demonstrate that our proposed method can remove batch effect effectively and outperform all compared methods in terms of classification accuracy. This is attributed to multiple modules that are interacted with each other in our framework. With regards to this, the multiple losses have taken effect through the calibrator $C$ and discriminator $D$, in which the former penalizes the domain mismatch for the different batches and the latter rewards the similarity for the same category in different batches. The joint learning of the two tasks can substantially improve the overall performance of our network.

A considerable number of computational methods have been developed for removing batch effect especially in the field of genomics and transcriptomics. However, they might be less effective on improving the accuracy of diagnosis based on MALDI MS based metabolomics. Taking ComBat as an example, the accuracy basically remains the same level as raw data in our experiments. The algorithm of fSVA is originally designed concerning the prediction or classification task, so it can yield better performance than ComBat or ratio_G. Although ResNet is based on deep learning, it is not superior to fSVA in accuracy, as shown in Table 5. A possible reason points to the fact that it doesn’t make full use of the supervision provided by source labels. As for NormAE, apart from distinguishing batch labels instead of biological labels during the adversarial process, the dimensionality of latent space may also be one of the reasons why it is inferior to us. Unlike its strategy, the number of nodes in hidden layers from input to output remains the same, although we also utilize the autoencoder backbone. According to our attempts, compressing the number of features in latent space will lead to a decline for classification performance. Overall, previous publications cannot effectively address batch effect especially concerning the need of classification.

There are many findings regarding the experimental results in this work. Given the classification results on the simulation study of CyTOF data, one may notice that the in-batch tenfold cross-validation for Patient 1 yields the AUC of 0.961. While batch effect is fully ruled out in the in-batch validation, the four metrics should be perceived as a ceiling of performance for cross-batch validation. However, when treating Day 2 as source and Day 1 as target, the cross-batch AUC is 0.968, even beyond the in-batch ceiling. This phenomenon might as well be caused by the imbalanced sample sizes of the two batches. In other words, the larger size of the training data will help build a more robust classifier despite of the batch effect.

Although our method achieves the best performance across all comparing metrics, there are still several deficiencies in this work that cannot be underestimated. The first is that we may not obtain perfect performance for each compared algorithm by enumerating all hyperparameters. Next, our work is applicable to single-source-single-target scenario only. In the presence of multiple batches that can be often encountered in real-world settings, it is highly expected that adapting a trained model to be used by multiple unlabeled target batches, or boosting the classification performance by using multiple source batches for training. Last but not least, beyond the fact that this approach is no longer unsupervised and requires domain knowledge, the amount of labeled data that might be needed to achieve reasonable performance can possibly be large. Future improvements will carry out from the perspectives of these items.

## Conclusions

The research of non-targeted metabolomics represents an important part of human physiological mechanism study as well as in the aspect of phenotypic disease. However, these human clinical studies often require multiple batches followed by some analytical barriers. Combining data from different batches without carefully removing batch effect can be strong enough to interfere the outcomes. Many approaches to adjust for batch effect have been developed, yet they focus more on batch effect removal rather than downstream classification.

Here we have introduced a novel joint deep learning framework for simultaneous batch calibration and classification. Our framework consists of three major parts – calibrator, reconstructor(s), and discriminator. Upon the simulation study on CyTOF data and case study on MALDI MS data, we find that our method can effectively eliminate batch effect and then complete classification, yielding significantly better performance than existing state-of-the-art methods. We have witnessed here applications to MALDI MS based metabolomics data and released publicly available code. This novel tool will produce great potential where other metabolomics (i.e., LC–MS) even other omics technologies are applied for the analysis of large samples in clinical studies practically.

## Methods

**Fig. 4:**
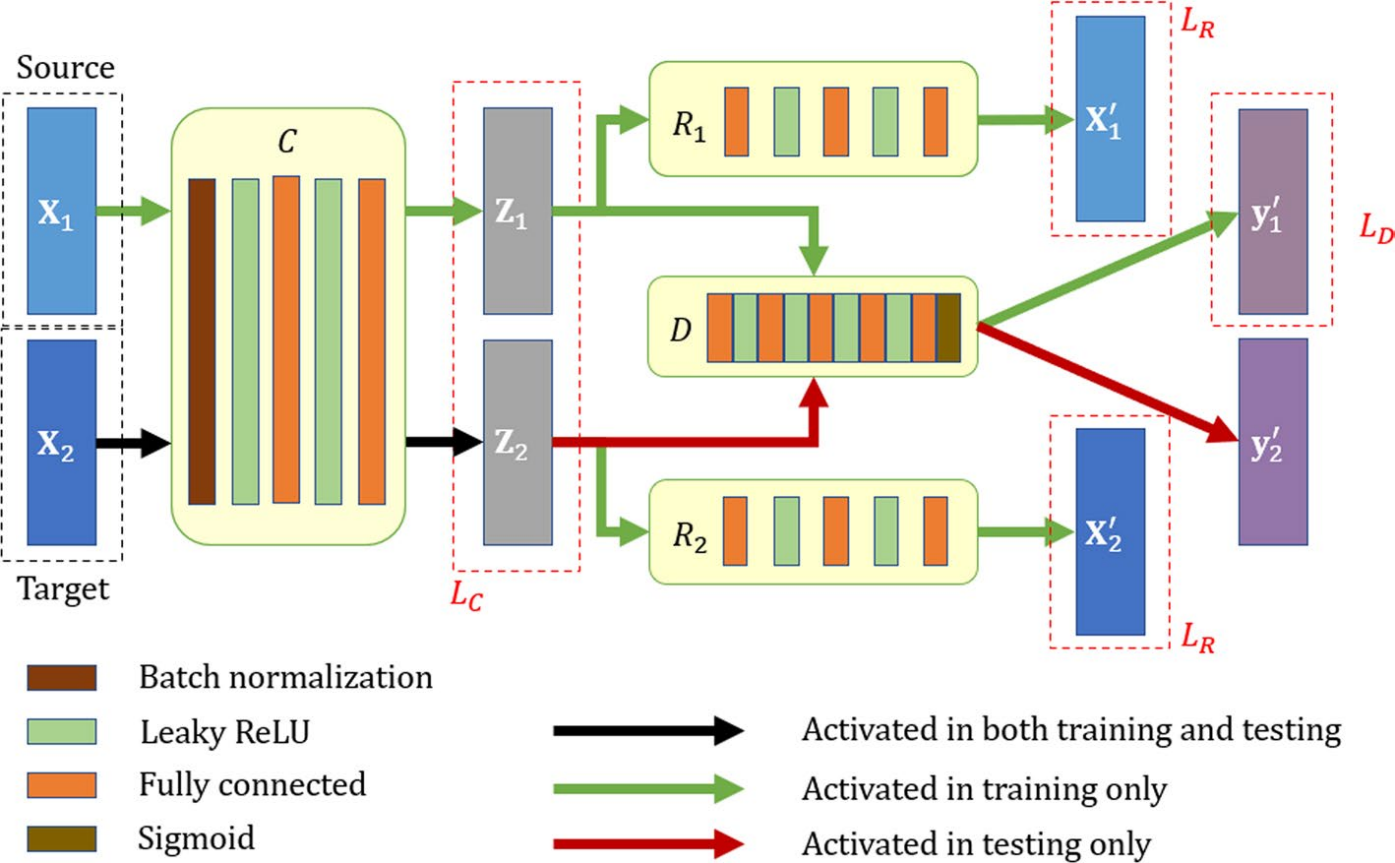
The architecture of the proposed deep learning framework for joint batch effect removal and classification. The source batch ${\mathbf{X}}_{1}$ and the target batch ${\mathbf{X}}_{2}$ are processed through the same calibrator ${\varvec{C}}$, such that both batches are compactly distributed in the latent space. The source batch supervises the training of the discriminator ${\varvec{D}}$, which then predicts the labels for the target batch in testing. Two reconstructors, ${\varvec{R}}_{1}$ and ${\varvec{R}}_{2}$, are used to ensure that the input data can be fully recovered from latent encoding

The application scenario of our method is to train a model from known labels of the source batch, such that the samples in the target batch can infer their labels by classification. We denote the source batch as the matrix ${\mathbf{X}}_{1}$ accompanied with the labels in the vector ${\mathbf{y}}_{1}$, and the ‘unlabeled’ (not input to $D$ during the training stage) target data is ${\mathbf{X}}_{2}$. Our goal is to find a calibrator $C$, such that the two calibrated batches, ${\mathbf{Z}}_{1} = C\left( {{\mathbf{X}}_{1} } \right)$ and ${\mathbf{Z}}_{2} = C\left( {{\mathbf{X}}_{2} } \right)$, distribute compactly in the latent space. The calibrated data then pass through the discriminator $D$, which produces the classification label per sample. Note that $D$ is only trained on the source batch with its corresponding labels, and then we leverage the well-trained discriminator to classify the calibrated samples in the target batch. To make sure that the latent space encodes powerful and well-functioning representation, we require all sample data to be fully reconstructed from the calibrated latent space, e.g., by passing through two individual reconstructors, $R_{1}$ and $R_{2}$, respectively. Figure 4 illustrates the overall framework of our method consisting of calibrator, reconstructor(s) and discriminator.

### Calibrator

Our calibrator is responsible for reducing the discrepancy between the source and target by mapping two batches of data to a common latent space. In order to facilitate the convergence in training, we enforce the first layer of the calibrator to be batch normalization (BN). The subsequent layers consist of two fully connected (FC) layers and two Leaky ReLU activation layers. The number of nodes in each hidden layer always remains equal to the dimensionality of the input, such that the length of the latent code per sample is not compressed with respect to the number of their original features.

The loss of the calibrator needs to measure the divergence between the source and the target distributions. Particularly, we train our calibrator to minimize the maximum mean discrepancy (MMD) between the two batches in the latent space

1 $${\mathcal{L}}_{C} = C\left( {{\mathbf{x}}_{1}^{\left( i \right)} } \right) - C\left( {{\mathbf{x}}_{2}^{\left( j \right)} } \right)_{1} ,$$

where ${\mathbf{x}}_{1}^{\left( i \right)}$ and ${\varvec{x}}_{2}^{{\left( {\varvec{j}} \right)}}$ indicate the $i$-th sample in the source batch ${\mathbf{X}}_{1}$ and the $j$-th sample in the target batch ${\mathbf{X}}_{2}$, and $\cdot_{1}$ is the L1-norm operator. The MMD measure vanishes when the underlying distributions are highly similar.

### Reconstructor

We expect the calibrated data to be free of batch effect, and to encode solely the intrinsic biological states in the samples (e.g., disease labels). To prevent losing the class semantics encoded in the latent code ${\mathbf{Z}}$, we introduce $R\left( {\mathbf{Z}} \right)$ to transform the latent code ${\mathbf{Z}}$ to the reconstructed ${\mathbf{X}}^{\prime}$. In other words, the reconstructor is responsible for mapping the latent code back to the original sample data.

The calibrator and reconstructor in overall form an encoder-decoder backbone for self-learning. There are three FC layers and two Leaky ReLU activation layers in each reconstructor. All the FC layers share the same initialization strategy and the number of nodes in the hidden layers always remains equal to the dimensionality of the input. The reconstruction loss is thus calculated as the residual in L2-norm between the reconstructed output and the input prior to calibration:

2 $${\mathcal{L}}_{R} = {\varvec{x}}^{{\left( {\varvec{i}} \right)}} {-}R\left( {C\left( {{\varvec{x}}^{{\left( {\varvec{i}} \right)}} } \right)} \right)_{2}^{2} .$$

Note that each reconstructor is corresponding to a certain batch.

### Discriminator

Finally, we derive $D$ as a task-specific label classifier. At training stage, the input data generates ${\mathbf{Z}}$ that can represent its semantics through the calibrator $C$, and then it is transmitted to the discriminator $D$ for classification. In the latent space, each encoded source sample ${\mathbf{z}}_{1}$ and target sample ${\mathbf{z}}_{2}$ are accompanied with their corresponding ground-truth class label and the to-be-predicted label. At inference stage, the well-trained $D$ is expected to achieve high discriminative ability on the target batch, under the supervision of the source batch.

The network of the discriminator has the similar specification but different numbers of layers with calibrator/reconstructors, namely five FC layers and four Leaky ReLU activation layers. The numbers of nodes in the five FC layers are reduced from 128 to 64, 32, 16 and finally to 1 (in that we only consider binary classification in our later experiments). The Sigmoid activation function is utilized by the last layer to complete classification.

We calculate the discriminator’s loss as binary cross entropy (BCE) between its output and samples’ labels. When there are only two categories to classify, the discriminator is trained by minimizing

3 $${\mathcal{L}}_{D} = - {\varvec{y}}_{1}^{\left( i \right)} \log D\left( {C\left( {{\varvec{x}}_{1}^{\left( i \right)} } \right)} \right),$$

where ${\varvec{y}}_{1}^{\left( i \right)}$ can be either 0 (for negative training sample) or 1 (for positive). The class label information of the test data is only used when evaluating the prediction performance and the information is unknown during the network training process.

### Implementation details

The total loss of our framework is then calculated by considering the calibrator, reconstructor(s), and discriminator as a whole, i.e., to minimize

4 $${\mathcal{L}} = \alpha \cdot {\mathcal{L}}_{R} + \beta \cdot {\mathcal{L}}_{C} + \gamma \cdot {\mathcal{L}}_{D} ,$$

where $\alpha$, $\beta$, $\gamma$ are scalar weights for each component network.

We implement the proposed solution with PyTorch (version 1.3.1) and Sklearn (version 0.21.3). The downstream analysis has been carried out using Python (version 3.6.8), and R (version 3.6.3) for visualization. For details, we use ADAM [33] for training with default settings (i.e., the exponential decay rate of the first/second moment estimation). All the experiments are run on the same host with 16 GB memory and an Nvidia RTX 2080Ti GPU.

## Supplementary Information

**Additional file 1:** Implementation details and supplementary results.

## Data Availability

The dataset supporting the conclusions of CyTOF experiment is included within the article. Complete code and the preprocessed version of our private MALDI MS metabolomics data are publicly available at: https://github.com/n778509775/JDLBER.git, and the original data that support the findings of this study are available from the corresponding author upon reasonable request.

## Abbreviations

MS: Mass spectrometry
MALDI: Matrix assisted laser desorption/ionization
SVA: Surrogate variable analysis
MMD: Maximum mean discrepancy
CyTOF: Cytometry by Time-Of-Flight
LS: Location-scale
MF: Matrix-factorization
SLE: Systemic lupus erythematosus
MCC: Matthews correlation coefficient
