# Supplementary material for: Joint deep learning for batch effect removal and classification toward MALDI MS based metabolomics
Source: BMC Bioinformatics. 2022 Jul 10;23:270. doi: 10.1186/s12859-022-04758-z (PMC9275160; doi:10.1186/s12859-022-04758-z)
Supplement: Supplementary file 1 — Additional file 1: Implementation details and supplementary results. [file 12859_2022_4758_MOESM1_ESM.pdf]

1 Versions or Parameters

1.1 CyTOF Dataset

1.1.1 Before calibration

In this study, we all use deep learning networks for classification. To achieve fair comparison, the types and numbers of network layers remain the same before and after calibration. The parameter “MALDI_MS” is a flag of mass spectrometry experiments, therefore it is set to False on all public CyTOF data. The learning rate, number of epochs, size of mini-batch and learning step are set to 10^{-4}, 100, 200 and 10^{4} respectively. In order to prevent overfitting the network, the L2 weight decay is set to 5×10^{-5} during training.

1.1.2 After calibration

For training our network after calibration, the learning rate is set to 10^{-4} and the number of epochs is set to 2000. The gradient update rule of mini-batch in deep learning is used to train our model where three losses are calculated from a sampled “mini-batch” during each iteration of the training process. We set the mini-batch size to 100 and the coefficients of three losses are $\alpha = 0.01$, $\beta = 1$, $\gamma = 0.01$ by grid-search. The learning step, L2 weight decay are set to 10^{4}, 5×10^{-5} during training.

1.1.3 In-batch Cross Validation

In order to compare all the results on the same benchmark, the classification network of in-batch 10-fold cross-validation shares the same framework as cross-batch experiments. The learning rate, number of epochs, size of mini-batch, learning step and L2 weight decay are set to 10^{-4}, 15, 200, 10^{3} and 5×10^{-5}, respectively.

1.2 MALDI MS Dataset

1.2.1 Before calibration

In this set of experiment, the flag variable “MALDI_MS” is set to True on all private MALDI MS data. The ID of the file containing the number of samples in each subject is consistent with testing set. The structure of the classification network is also consistent with the CyTOF experiments. The learning rate, number of epochs, size of mini-batch, learning step and L2 weight decay are set to 10^{-3}, 100, 200, 10^{4} and 5×10^{-5}, respectively.

1.2.2 After calibration

For training after calibration, the ID of the file containing the number of samples in each subject is consistent with testing set. The learning rate, number of epochs, size of mini-batch and learning step are set to 10^{-4}, 2000, 200 and 10^{4}, respectively. In addition, the hyper-parameters of the coefficients of three losses are set to $\alpha = 0.01$, $\beta = 1$, $\gamma = 0.01$ by grid-search. The L2 weight decay is set to 5×10^{-5} during training, the same with first experiment.

1.2.3 In-batch Cross Validation

As the previous experiment, the classification network of in-batch cross-validation holds the same structure as cross-batch predictions. The ID of the file containing the number of samples in each subject is consistent with training set. The learning rate, number of epochs, size of mini-batch, learning step and L2 weight decay are set to 10^{-4}, 25, 200, 10^{3} and 5×10^{-5}, respectively.

1.3 Information for Other Methods

Corresponding open source code could be found about those algorithms involved in comparative experiments. The ComBat and fSVA have been implemented by ComBat() and fsva() function respectively into R software package sva (http://bioconductor.org/packages/3.5/bioc/html/sva.html). In addition, commonly used batch effect removal functions including geometric.mean() that ratio_G adopted is implemented in Psych R package (http://cran.r-project.org/web/packages/psych/index.html). In principle, ratio-based data is obtained by scaling all samples through ready-made reference samples or the average of negative class samples in each batch. However, it should be mentioned that the two means above are not available in practice, because it is not possible to know the class label of the test batch before performing the prediction. We choose to utilize the mean of whole train samples (namely batch 1) as the reference and scale other batch sample values (intensity) by it, thus not leading to significant performance bias. The source codes of ResNet and NormAE algorithm are publicly available at https://github.com/ushaham/BatchEffectRemoval.git and https://github.com/luyiyun/NormAE, respectively. Since our data based MALDI MS instead of LC MS in NormAE, which not exist so-called injection order, therefore, it is eliminated in training and testing process. In addition, the mass quality control was conducted using standard molecules on the stage of serum plates, so it doesn’t appear at the preprocessing matrix. In order to ensure the convergence of the model, except for the lr_disc_b, epoch and batch_size which are set to 0.0005, (100, 10, 100) and 200, other parameters are defaulted.

2 Additional Visualization Results

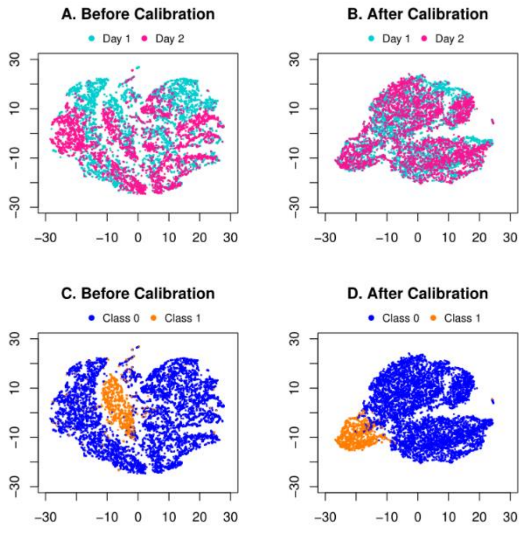

Fig. S1. Visualization of Patient 2 of the public CyTOF data. This illustration has exactly the same situation as the caption of Figure 1. For details, please refer to the explanation in Fig. 1.

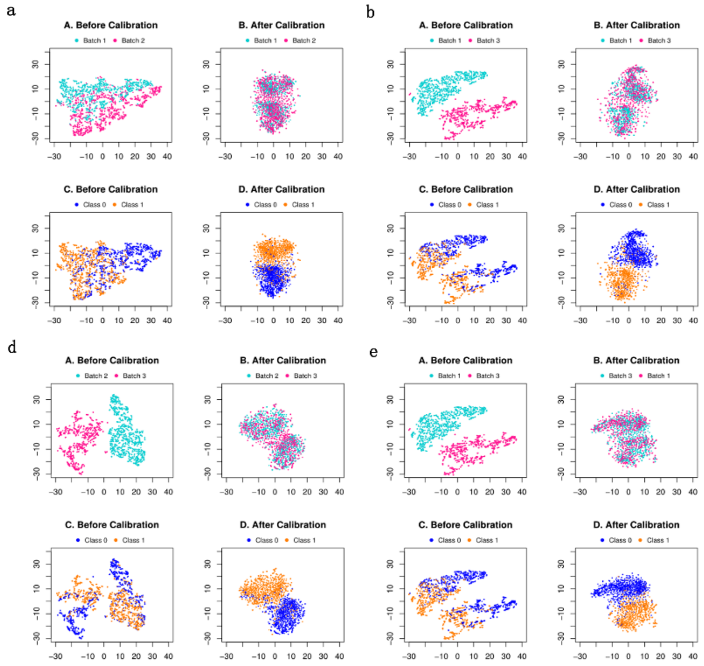

Fig. S2. Visualizations of remaining five groups in private MALDI MS data. Its situations and legend explanations are the same as caption in Fig. 2. **a** Source: batch 1; Target: batch 2. **b** Source: batch 1; Target: batch 3. **c** Source: batch 2; Target: batch 1. **d** Source: batch 2; Target: batch 3. **e** Source: batch 3; Target: batch 1.

3 Robustness Verification

To prove the deep learning algorithm we developed could be applied to an entirely new similar set, we have collected a new batch of systemic lupus erythematosus (SLE) patients and healthy controls (HCs) subjects from Renji Hospital, including 89 SLEs and 75 HCs. We utilize the previously trained three batches to calibrate the batch effect of this new batch and predict its ACC, F-score, AUC and MCC. As shown in Table S1, no matter which batch is used for the training set, these indicators can be significantly improved after calibration.

Table S1. Cross-batch prediction results of original three batches as source and the new batch as target

Source Batch		Sample Level			Subject Level		
		1	2	3	1	2	3
ACC	Before	0.773	0.810	0.755	0.768	0.805	0.756
	After	0.884	0.837	0.872	0.878	0.835	0.872
F-score	Before	0.806	0.831	0.723	0.802	0.826	0.726
	After	0.897	0.837	0.874	0.891	0.834	0.874
AUC	Before	0.764	0.805	0.770	0.759	0.800	0.771
	After	0.880	0.842	0.877	0.874	0.842	0.877
MCC	Before	0.544	0.616	0.568	0.534	0.606	0.567
	After	0.767	0.684	0.752	0.755	0.685	0.752

Permutation test is a computationally intensive based method that utilizes random arrangement of sample data for statistical inference. If random or “fake” labels could not achieve good results, our model is robust. Otherwise, it means that there are some problems with the model. Taking one old batch (i.e., batch 1) for training and the new batch for test, our implementation is the same shuffling pattern as the old batches in Figure 3. The accuracy values of random label are bell-shaped and very poor (Figure S3a). In addition, we have also tried to quest whether the differences of key metabolites are still significant after arranging the measurement for this new batch. As shown in Figure S3b, the results illustrate that there is no significant difference in any m/z feature between the case and control groups, which further verifies the robustness of the algorithm in our study.

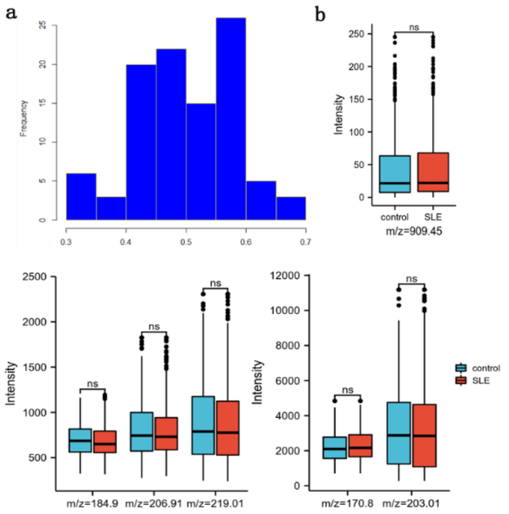

Fig. S3. **a** Permutation test of accuracy for old batch (batch 1) as source and new batch as target. **b** Boxplots of those six common m/z features on the condition of permutating the labels in the new batch.
